# Prognostic Value and Potential Mechanism of MTFR2 in Lung Adenocarcinoma

**DOI:** 10.3389/fonc.2022.832517

**Published:** 2022-05-05

**Authors:** Zengzhi Lian, Pei Pang, Yan Zhu, Wenwen Du, Jintao Zhou

**Affiliations:** ^1^ Department of Pulmonary and Critical Care Medicine, Taicang Affiliated Hospital of Soochow University, Suzhou, China; ^2^ Department of Pathology, The First Affiliated Hospital of Soochow University, Suzhou, China; ^3^ Department of Emergency and Critical Care Medicine, Changzheng Hospital of Second Military Medical University, Shanghai, China; ^4^ Department of Pulmonary and Critical Care Medicine, The First Affiliated Hospital of Soochow University, Suzhou, China

**Keywords:** MTFR2, prognosis, immunity, cell proliferation, lung adenocarcinoma

## Abstract

Mitochondrial fission regulator 2 (MTFR2) belongs to the MTFR1 family, which plays a crucial role in regulating oxidative phosphorylation. Recent studies indicate that it also participates in cancer carcinogenesis and development; however, the clinical significance of MTFR2 in lung adenocarcinoma has not been fully confirmed. Our current study investigated the relationships between clinical characteristics and MTFR2 expression based on The Cancer Genome Atlas (TCGA), Gene Expression Omnibus (GSE31210) dataset, and clinical histopathological sample cohort. In addition, Kaplan–Meier and Cox regression analyses were additionally performed to evaluate the association between MTFR2 expression and patient survival. Gene set enrichment analysis (GESA) was conducted to spot possible pathways associated with MTFR2. Moreover, a single-sample GESA (ssGESA) was performed to evaluate the association between MTFR2 expression and immune cell infiltration. Cell colony formation assay, CCK-8 assay, cell cycle assay, and transwell assay were performed to verify the cell proliferation, migration, and invasion abilities after interfering with MTFR2 in lung cancer cells. Western blot assay was applied to identify the underlying protein levels. The results indicated that the elevated MTFR2 expression in lung adenocarcinoma samples correlated with T stage (*P* < 0.001), N stage (*P* = 0.005), M stage (*P* = 0.015), pathological stage (*P* = 0.002), and TP53 status (*P* < 0.001). Patients with a higher MTFR2 expression correlated with poorer overall survival (*P* < 0.01) and progression-free survival (*P* = 0.002). Knockdown of MTFR2 inhibited cell proliferation, migration, and invasion *via* AKT-cyclin D1 signaling and EMT pathways. Moreover, MTFR2 expression significantly positively correlated with Th2 cells (*P* < 0.001). Taken together, MTFR2 could serve as a novel prognostic indicator and therapeutic target for lung adenocarcinoma.

## Introduction

Over the past years, there has been a tremendous increase in lung cancer-related deaths worldwide (1, 2). Although significant advances have been made in lung cancer therapy, the 5-year survival rate of lung cancer is still less than 20% (3). Lung adenocarcinoma (LUAD) is the primary cause of lung cancer-associated death. Accumulated clinical trials and investigations have been made to explore the pathogenesis in lung adenocarcinoma development. However, the molecular mechanism remains unclear. Therefore, there is an urgent need to explore more reliable molecular biomarkers in LUAD-associated tumor prognosis, carcinogenesis, and development.

Mitochondria are the place where glucose, glutamine, and lipid metabolism takes place (4). Mitochondrial fusion and fission determine the shape of the mitochondrial network and ensure homeostatic maintenance. The mitochondria mainly function to generate adenosine triphosphate (ATP) (5). Mitochondrial dysfunction, including mitochondrial oxidative phosphorylation system (OXPHOS) defects, mitochondrial DNA mutations, and alterations of the mitochondrial genome, may lead to the occurrence of multiple types of human cancers (6–8). In addition, alterations of the electron transport chain can affect cancer cell metabolism, apoptosis, and drug resistance (9, 10). Mitochondrial fission regulator 2 (MTFR2) is located on the 6q23.2 chromosome, which can be generated by alter splicing. MTFR2 can promote mitochondrial fission and regulate glucose metabolism. Previous studies have confirmed that MTFR2 plays a great part in aerobic respiration (11, 12). However, recent studies have also shown that MTFR2 was associated with patient age, tumor TNM stage, molecular subtype, and grade, indicating poorer prognosis (13, 14). In glioblastoma, MTFR2 can transcriptionally regulate TTK expression in maintaining glioma stem-like cells (12). In breast cancer, MTR2 can promote cell growth, migration, and invasion and switch glucose metabolism from OXPHOS to glycolysis in an HIF1α- and HIF2α-dependent manner (15). Similar phenomena were also found in oral squamous carcinoma (16). In gastric cancer, MTFR2 expression was significantly correlated with the infiltration levels of CD8^+^ T cells, CD4^+^ T cells, macrophages and dendritic cells (17). Moreover, for lung cancer, limited bioinformatics studies have demonstrated that MTFR2 was a biomarker for diagnosis and poor prognosis in LUAD (18). Furthermore, the detailed expression levels and function in LUAD have not been fully investigated. Herein, our current study intended to clarify the roles of MTFR2 in LUAD and its clinical pathological characteristic, immune infiltration, and prognosis, which implies MTFR2 as a prognosis biomarker for LUAD patients.

## Materials and Methods

### Data Collection and Analyses

In the current study, gene expression data (HTSeq-FPKM) and clinical information of 535 LUAD patients were downloaded from UCSC Xena (https://xenabrowser.net/datapages/). Then, the level 3 HTSeq-FPKM data were transformed into TPM (transcripts per million reads) for further analysis (19). Clinical data included in this study are age, gender, race, TNM stage, primary therapy outcome, residual tumor, anatomic neoplasm subdivision, P53 status, KRAS status, and smoke status. GEO (https://www.ncbi.nlm.nih.gov/geo/) dataset GSE31210 which consisted of 226 patients was also included in this study.

### Gene Set Enrichment Analysis

We performed GSEA (gene set enrichment analysis) by utilizing the R package and clusterProfiler to show the differential pathways between the high- and low-MTFR2 expression groups (20). Gene set permutations were performed 1,000 times for each analysis. The pathways significantly enriched were adopted as adjusted P-value of <0.05, False discovery rate (FDR) q-value of <0.25 and normalized enrichment score(NES)>1.0.

### Immune Infiltration Analysis by Single-Sample Gene Set Enrichment Analysis

We performed an immune infiltration analysis using ssGSEA (single-sample gene set enrichment analysis) of the GSVA package *via* R software (version 3.6.2) (21). The levels of the 24 tumor-infiltrated immune cell types were calculated. The correlation between MTFR2 and the infiltration levels of immune cells was resolute by calculating the Spearman correlation. The Wilcoxon rank-sum test was adopted to analyze the association between the different expression levels of MTFR2 and the infiltration of immune cells.

### Construction of the Nomogram

A nomogram was built by the rms R package (version 3.6.3) based on multivariate analysis. The quality of the nomogram model was confirmed by the concordance index (C-index). The prediction accuracy of the nomogram depends on the value of the C-index.

### Hematoxylin–Eosin Staining and Immunohistochemical Assay

#### Patient Samples

A total of 40 paired LUAD tissue specimens were collected in the Department of Respiratory Medicine, Taicang Affiliated Hospital of Soochow University. All participants were provided with written informed consent at the time of recruitment. All samples were kept at -80°C for storage. All cases had clinically and pathologically confirmed diagnoses of LUAD based on the Revised International System for Staging Lung Cancer. Ethics approval was obtained from the local Institutional Review Board committee. Paraffin-embedded tissues were sectioned into 5-μm slides and mounted. Slides were deparaffinized, rehydrated with ethanol, and quenched in 30% vol/vol hydrogen peroxide/methanol (1:9) for 15 min. Non-specific antigens were blocked by incubating in 2% BSA and 0.1% Triton X-100 for 30 min at room temperature. The sections were incubated with primary antibodies anti-MTFR2 (1:100 dilution; Sigma-Aldrich, St. Louis, Missouri, USA) and anti-GATA3 (1:100 dilution, Santa Cruz, Dallas, Texas, USA) overnight, followed by a secondary antibody, then stained with diaminobenzidine (DAB, Vector Laboratories, Burlingame, CA) and hematoxylin (nuclei stain). ImageScope software was used to quantify the intensity and positively stained areas. Two pathologists independently scored immunohistochemistry staining.

### Cell Culture and Transfection

A549 and H1299 cells were cultured in RPMI 1640 medium supplemented with 10% FBS (fetal bovine serum, Gibco, Grand Island, NY, USA) and 1% penicillin/streptomycin. Cells were seeded onto a 6-well plate before transfection. When the cell density reached 40%–50%, si-MTFR2-1, si-MTFR2-2, or negative control mixed with the jetPRIME transfection reagent (Polyplus-transfection, Ozyme, Paris, France) were added into cells according to the manufacturer. All siRNAs were purchased from RiboBio Company. The detailed sequences of siRNAs targeting MTFR2 are as follows: siRNA-MTFR2-1: 5′-CTAGGAGTATTGTTCGTAT-3′ (stB0014895A); siRNA-MTFR2-2: 5′-GTACAACCAGGATCTAATA-3′ (stB0014895B).

### CCK-8 Assay and Cell Colony Formation Assay

Cell viability was detected by the Cell Counting Kit-8 (Beyotime, Shanghai). After transfection with si-RNAs, cells were seeded into 96 wells at 3,000 cells each well and cultured for 24, 48, and 72 h. Then, 10 μM of CCK-8 reagent was added and incubated for additional 2 h; absorbance was measured at 450 nm. Moreover, for cell colony formation assay, the above cells were collected and placed onto 6-cm dishes (1,000 cells per dish) for 10–14 days. Then, cells were washed with PBS, fixed with 100% methanol, and stained with 0.1% crystal violet. Colony numbers were counted and graphed.

### Quantitative Real-Time PCR Analysis

After transfection, A549 and H1299 cells were collected, and RNA was extracted by TRIzol Reagent (TaKaRa, Kyoto, Japan) and subjected to reverse transcription using the TaqMan reverse transcription kit (Takara, Japan). The primers used were as follows: MTFR2, 5′-GAAACTGGATCCCAATGTGAA-3′ (forward) and 5′-GAATAAGGTTAAGCTTCGTGCAA-3′ (reverse); and GAPDH 5′-TCTGGTAAAGTGGATATTGTTG-3′ (forward) and 5′-GATGGTGATGGGATTTCC-3′ (reverse). Quantitative real-time PCR analysis was measured using an ABI Prism 7000 Sequence Detection System (Applied Biosystems, Foster City, CA) with SYBR Green PCR SuperMix (TaKaRa, Japan). All samples were assessed in triplicate.

### Cell Cycle Analysis

After being transfected with si-MTFR2 for 48 h and treated with sc79 (5 μM, Beyotime, CA) for 2 h, cells were centrifuged and washed in PBS, then resuspended in 75% ethanol overnight at 4°C. After being incubated with RNAse + propidium iodide + sodium citrate (50 mM) for 30 min at 37°C, the cells were analyzed by flow cytometry (BD LSRFortessa, Franklin Lakes, NJ, USA). To analyze the proportion of cells in G1, S, and G2/M, the FlowJo cell cycle Watson (Pragmatic) model with the G2 peak constrained on G1 = G2 × two was used.

### Transwell Assay

The 8-μm-pore-size polycarbonate membrane was pre-incubated with (invasion assay) or without 1:4 DMEM-diluted Matrigel (migration assay) (Corning, CA) for 2 h. Then, cells were centrifuged, and 4 × 10^5^ cells diluted in 1% medium were added to the upper chamber of the transwell (Falcon, CA). The lower chamber was then filled with 10% medium. After incubation at 37°C for 24 h, cells were fixed with methanol and stained with 0.2% crystal violet. Cells at the top side were removed with a cotton swab, then cells at the bottom side were observed, with images taken, and counted.

### Western Blot Assay

Cells were lysed with RIPA lysis buffer (Beyotime, CA) for 30 min on ice and centrifuged at 12,000 g for 15 min at 4°C. The protein concentration was determined by a BCA protein assay kit (Beyotime, CA). Primary antibodies applied for Western blot were as follows: anti-MTFR2 (1:500, Sigma-Aldrich, HPA029792), anti-cyclin D1 (1;1,000, Beyotime, AF1183), anti-AKT (1:1,000, Cell Signaling Technology, #4685), anti-p-AKT(1:1,000, Cell Signaling Technology, Ser473, #4060), and anti-GAPDH (1:1,000, Abcam, ab181602). Wb analysis was performed using primary antibodies and corresponding secondary antibodies. After incubation with secondary antibodies, the members were detected using ECL Chemiluminescent Substrate Reagent Kits (Beyotime, CA) and a ChemiDoc XRS+ System (Bio-Rad, Hercules, CA, USA).

### Statistical Analysis

For public database analysis, the Kruskal–Wallis test, Wilcoxon signed-rank test, and Spearman correlation were applied to analyze the correlation between clinical-pathological features and the expression of MTFR2. Univariate Cox regression analysis and the Kaplan–Meier method were applied to evaluate the prognostic factors. Then, multivariate Cox analysis was performed to compare the influence of different expression levels of MTFR2 expression with other clinical parameters. GraphPad Prism 7.0 (GraphPad, San Diego, CA, USA) and SPSS 17.0 software (SPSS, Chicago, IL, USA) were used for experimental calculations. All data were presented as mean ± SD. A non-paired Student’s t-test assessed significant differences between the two groups. Significant differences between three or more groups were analyzed using one-way or two-way ANOVA analysis followed by Bonferroni test. All statistical tests were two-tailed. *P* < 0.05 was set as statistically significant.

## Results

### MTFR2 Expression in LUAD Patients

**Figure 1:**
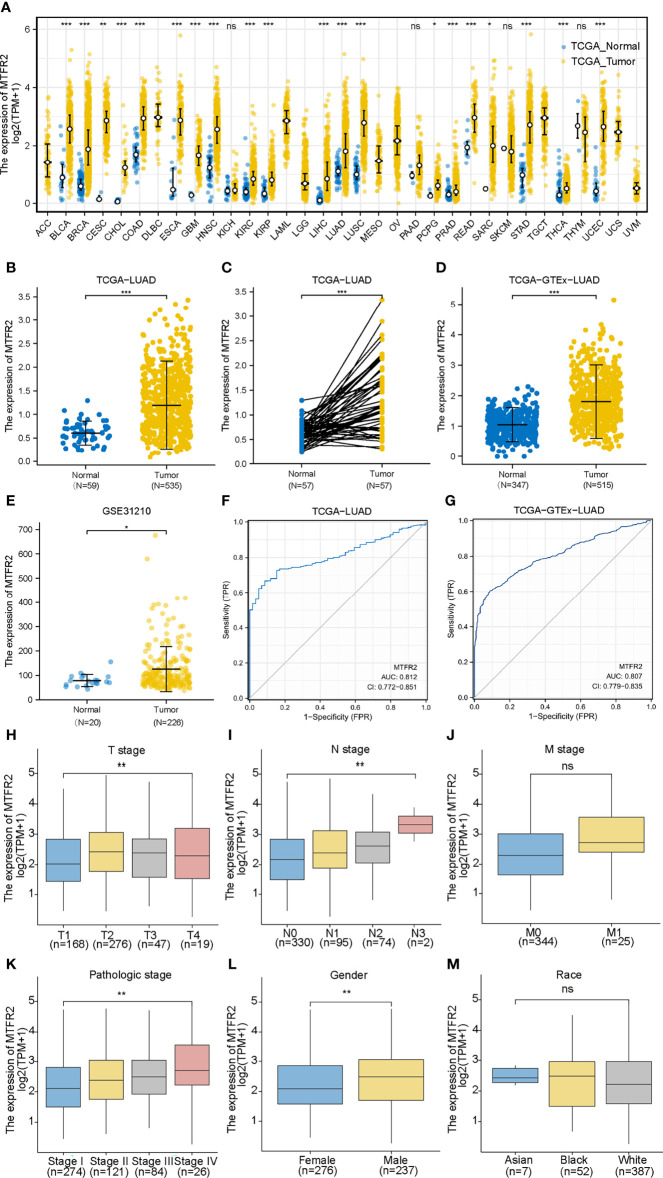
MTFR2 was highly expressed in LUAD tissues based on bioinformatical analysis. MTFR2 was overexpressed in pan-TCGA cancer **(A)**, TCGA-LUAD **(B)**, TCGA-LUAD paired samples **(C)**, TCGA-GTEx-LUAD **(D)**, and GSE31210 dataset **(E)**. ROC indicated that MTFR2 predicted the poor prognosis in TCGA and TCGA+GTEx data sets and AUC was 0.812 and 0.807 separately **(F, G)**. MTFR2 expression associated with clinic-pathological features: T stage **(H)**, N stage **(I)**, pathological stage **(K)**, and gender **(L)** but not significantly with M stage **(J)** and race **(M)**. LUAD, lung adenocarcinoma; ROC, received operation curve. *P < 0.05, **P < 0.01, ***P < 0.001, ns, no statistical significance.

**Figure 2:**
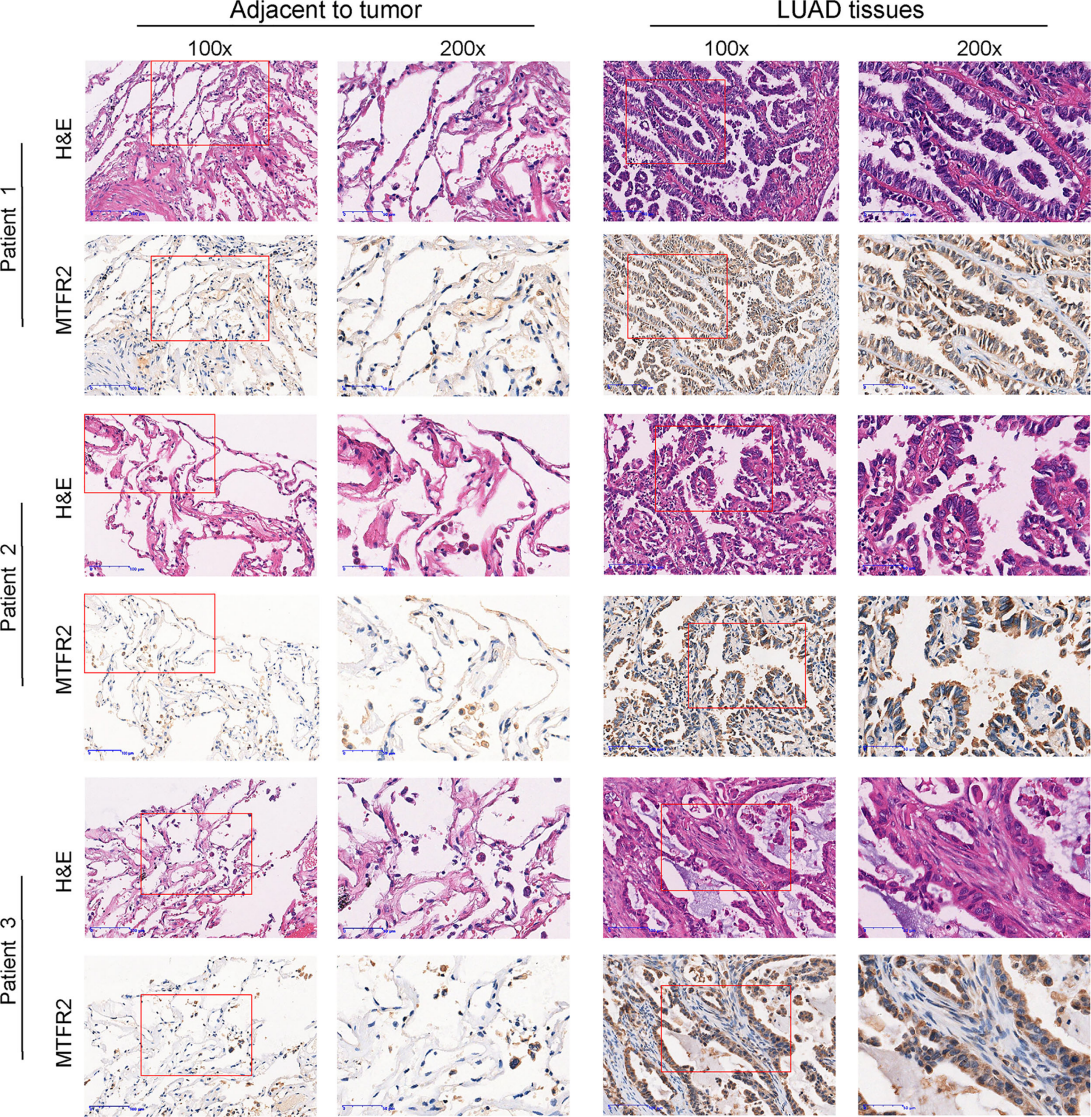
MTFR2 was overexpressed in LUAD patients when compared to adjacent samples *via* IHC and H&E assay (×100, ×200).

Based on the data extracted from TCGA-pan CANCER, TCGA-LUAD, and TCGA-GTEx-LUAD, we found that the MTFR2 expression levels were increased in TCGA pan-cancer and LUAD patients when compared to paired normal samples (**Figures 1A**–**D**). The GSE31210 dataset also showed a higher MTFR2 expression in lung cancer tissues (**Figure 1E**). The AUC values for MTFR2 in TCGA-LUAD and TCGA-GTEx-LUAD were 0.812 (CI: 0.772–0.851) and 0.807 (CI: 0.779–0.835), respectively (**Figures 1F, G**). Next, we extended our analysis to analyze the correlation between MTFR2 expression and clinical characteristics. The expression level of MTFR2 was considerably related to T stage (P = 0.007), N stage (P = 0.002), pathological stage (P = 0.003), and gender (P = 0.006) but not associated with M stage (P = 0.053) and race (P = 0.722) (**Figures 1H**–**M**). Moreover, immunohistochemical (IHC) assay similarly demonstrated that MTFR2 expression was elevated in LUAD patient samples (**Figures 2**). These results revealed that MTFR2 was overexpressed in LUAD tissues, which may contribute to LUAD progression.

### Clinical Characteristics Based on TCGA and GEO

As shown in **Table S1**, the lung adenocarcinoma patients’ clinical characteristics including TNM stage, pathologic stage, primary therapy outcome, gender, race, anatomic neoplasm subdivision, smoking history, TP53, and KRAS status were gathered. Among 513 patients analyzed in this study, 276 were women and 237 were men. Among these patients, 387 patients (86.8%) were white, 52 (11.7%) were black or African American, and 7 (1.6%) were Asian. In terms of TNM stages, 274 patients (54.3% ) were in stage I, 121 patients (24% ) in stage II, 84 patients (16.6%) in stage III, and 26 patients (5.1%) in stage IV. For the primary therapy outcome 204 of 315 patients, patients (73.9%) were CR, 68 patients (16%) were PD, 6 patients (1.4%) were PR, and 37 patients (8.7%) were SD cases. The anatomic neoplasm subdivision of the patients was determined as left opacity in 199 patients (40%), right opacity in 299 patients (60%), central lung in 62 patients (32.8%), and peripheral lung in 127 patients (67.2%). A total of 425 patients (85.2%) were smokers, while 74 (14.8%) were non-smokers. Patients with the TP53 mutant were 241 cases (47.4%), while the KARS mutant was found in 139 patients (27.4%). In addition, we found a higher expression of MTFR2 significantly associated with T stage (*P* = 0.001), N stage (*P* = 0.01), M stage (*P* = 0.012), pathological stage (*P* = 0.005), gender (*P* = 0.001), TP53 status (*P* < 0.001), age (*P* = 0.015), and number pack-years smoked (*P* = 0.005) (**Table S2**).

### Association Between MTFR2 and Survival

**Figure 3:**
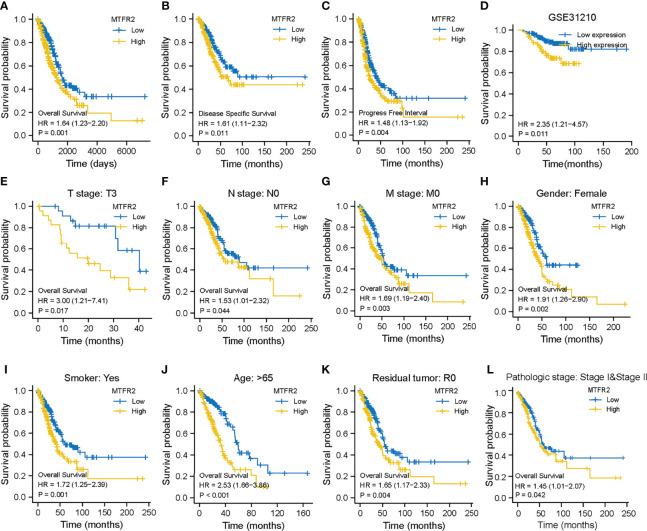
MTFR2 expression was correlated with survival status in LUAD patients. Higher MTFR2 expression was associated with poorer overall survival (*P* = 0.001) **(A)**, disease-specific survival (*P* = 0.011) **(B)**, progression-free interval (*P* = 0.004) **(C)**, overall survival (*P* = 0.011) **(D)**, and overall survival in T3 stage (*P* = 0.017) **(E)**, N0 stage (*P* = 0.044) **(F)**, M0 stage (*P* = 0.003) **(G)**, female gender (*P* = 0.002) **(H)**, smoke (*P* = 0.001) **(I)**, >65 years (*P* < 0.001) **(J)**, residual tumor R0 (*P* = 0.004) **(K)**, and pathological stage (*P* = 0.0042) **(L)**.

Kaplan–Meier survival analysis found that a higher MTFR2 expression in LUAD tissues was related to poorer overall survival, progression-free survival, and disease-free interval (**Figures 3A**–**C**). The GSE31210 dataset also indicated that MTFR2 overexpression was associated with reduced OS (**Figure 3D**). Subgroup survival analyses revealed elevated MTFR2 expression correlated worse survival with clinical features including T3 stage, N0 stage, M0 stage, female, smoker, >65 years, residual R0 tumor, and pathologic stage (**Figures 3E**–**L**). Univariate analysis showed that a high MTFR2 expression was linked to a poor OS (hazard ratio [HR]: 1.668; 95% confidence interval [CI]: 1.242–2.239; *P* < 0.001). As shown in **Figure S1A**, a higher MTFR2 expression analyzed by logistic regression was significantly correlated with poor prognostic factors, including T stage (OR = 2.11 (1.45–3.09) for T2, T3, and T4 vs. T1, *P* < 0.001), N stage (OR = 1.71 (1.18–2.49) for N1, N2, and N3 vs. N0, *P* = 0.005), M stage (OR = 3.20 (1.32–8.97) for M1 vs. M0, *P* = 0.015), pathological stage (OR = 1.74 (1.22–2.48) for stage II, stage III, and stage IV vs. stage I, *P* = 0.002), and TP53 status (OR = 6.18 (4.23–9.14) for Mut vs. Wildtype (WT), *P* < 0.001). The multivariate analysis results further found that MTFR2 was an independent indicator of poor OS (HR, 1.669; CI: 1.103–2.525, *P* = 0.015) and primary therapy outcome (HR, 3.185; CI: 2.094–4.845, *P* < 0.001) (**Figure S1B**). A nomogram was built based on multivariate analysis, including MTFR2 expression and primary therapy outcome (**Figure S1C**). The C-index value was 0.669 for MTFR2.

### MTFR2 Promote LUAD Cell Proliferation and Cell Cycle *via* the AKT Signaling Pathway

**Figure 4:**
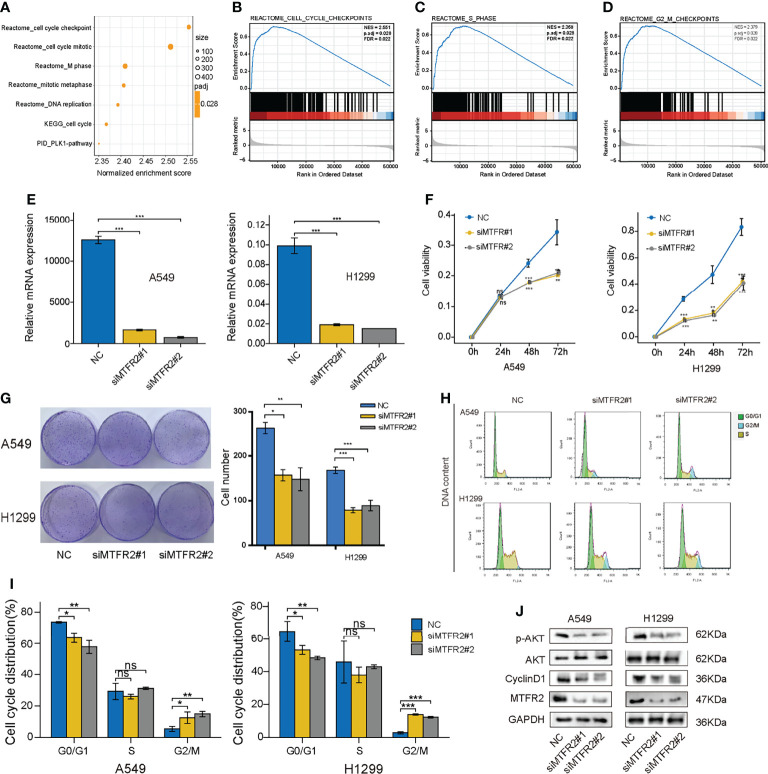
Knockdown of MTFR2 inhibited cell proliferation *via* the AKT signaling pathway. GSEA analysis showed that a high MTFR2 expression was associated with the cell cycle-related pathway **(A–D)**. qRT-PCR indicated that MTFR2 expression levels were decreased after knockdown of MTFR2 in A549 and H1299 cells **(E)**. CCK-8 assay **(F)** and cell colony **(G)** showed that cell proliferation were inhibited. Cell cycles were arrested in A549 and H1299 cells **(H, I)**. Western blot assay indicated that knockdown of MTFR2 downregulated the p-AKT and cyclin D1 expression levels **(J)**. *P < 0.05, **P < 0.01, ***P < 0.001, ns, no statistical significance. ES, enrichment score; NES, normalized ES.

Next, we intended to explore the potential role of MTGFR2 in LUAD progression. GSEA results showed considerable differences (FDR <0.05, adjusted P < 0.05) in the enrichment of the cell cycle checkpoints, cell cycle mitotic phase, M phase, and DNA replication pathway between the high MTFR2 expression and low MTFR2 expression groups (**Figures 4A–D**). In addition, a cellular response to external stress, RHO GTPase, and DNA repair signal was also enriched in the MTFR2-overexpressed group (**Figure S2**). To validate the above findings, A549 and H1299 were knocked down with two specific si-RNAs targeting MTFR2 (**Figure 4E**). MTFR2 knockdown resulted in cell growth inhibition *via* CCK-8 and cell colony formation assays (**Figures 4F, G**). The flow cytometry results showed that knockdown of MTFR2 expression resulted in a markedly decreased percentage of cells in the G0/G1 phases, whereas the percentage of cells in the G2/M phase was increased (**Figures 4H, L**). Finally, we go deeper into the potential mechanism beyond MTFR2-knockdown cells and found that p-AKT and Cyclin D1 expression was decreased (**Figure 4J**). These results suggested that MTFR2 can regulate LUAD cell proliferation and cell cycle *via* the AKT/Cyclin D1 signal pathway.

### The AKT Agonist Partly Reversed the Inhibitory Effect After Knockdown of MTFR2

**Figure 5:**
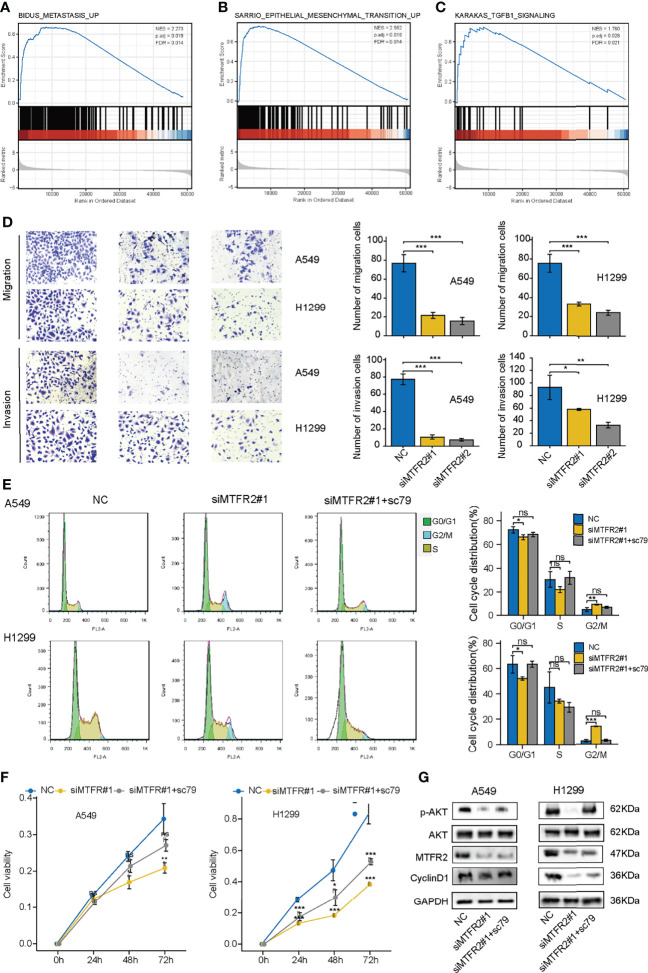
sc79 partly reversed the inhibitory effect after knockdown of MTFR2 in LUAD cells. GSEA analysis showed that MTFR2 overexpression was highly correlated with metastasis **(A)**, epithelial mesenchymal transition **(B)**, TGFB1 signaling **(C)**. Transwell assay revealed that knockdown of MTFR2 could decrease migrated and invasive ability of cancer cells **(D)**. sc79 could reverse cell cycle arrest **(E)** and inhibited proliferation **(F)** caused by knockdown of MTFR2, and relevant protein levels were upregulated **(G)**. *P < 0.05, **P < 0.01, ***P < 0.001, ns no statistical significance. ES, enrichment score; NES, normalized ES.

Apart from cell viability, we also explored the role of MTFR2 in regulating cell metastasis. As shown in **Figures 5A**–**C**, GSEA results showed that cell metastasis, epithelial–mesenchymal transition, and TGF-β1 signaling pathway were also enriched. Transwell assay showed that the migrated and invasive cells decreased after MTFR2 knockdown (**Figure 5D**), implying that MTFR2 also participates in LUAD cell metastasis. We also treated cells with SC79, an AKT agonist, to examine whether the activation of AKT signaling could reverse the inhibitory effect of MTFR2 knockdown on lung cancer cells. Flow data and CCK-8 assay showed that the sc79 agonist could increase the percentage of cells in the G2/M phase and partially reverse the suppressive effect on cell proliferation (**Figures 5E, F**). Western blot showed that knockdown of MTFR2 inhibited the activation of the AKT pathway and SC79 can partially reduce si-MTFR2-induced decreased p-AKT and Cyclin D1 expression, suggesting that MTFR2 could regulate lung cancer proliferation through the AKT signaling pathway (**Figure 5G**).

### The Correlation Between MTFR2 Expression and Immune Infiltration

**Figure 6:**
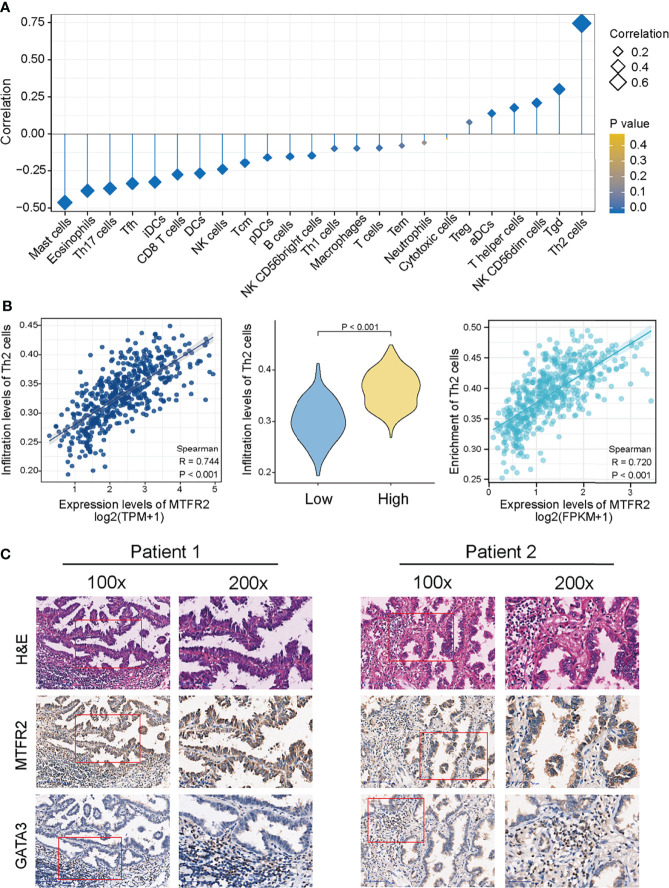
MTFR2 overexpression was correlated with the immune infiltration in the tumor microenvironment. The forest plot showed the correlation between MTFR2 expression and 24 immune cell subsets **(A)**. Correlation between the Th2 cells and the expression level of MTFR2 **(B)**. Immunochemistry showed the correlation of MTFR2 expression and Th2 cell biomarker GATA3 **(C)**.

The above finding pointed out that MTFR2 can contribute to tumor cell proliferation and metastasis. However, in addition to tumor cells, the tumor microenvironment also consisted of various immune cells. So next, we analyzed the correlation between the MTFR2 expression and immune cell infiltration (quantified by ssGSEA) by calculating the Spearman correlation. As shown in **Figure 6A**, MTFR2 expression was positively related to Tregs, aDC cells, T helper cells, NK CD56^dim^ cells, Tgd cells, and Th2 cells. Among all these cells, Th2 cells showed a significant correlation and were notably superior to other cells (Spearman r up to 0.744 or 0.720) (*P* < 0.001) (**Figure 6B**). Other immune cells including γδ T cells (r = 0.302, *P* < 0.001), NK CD56^dim^ cells (r = 0.21, *P* < 0.001), T helper cells (r = 0.176, *P* < 0.001), and aDCs (r = 0.139, *P* = 0.002) were significantly positively associated with MTFR2 expression, while mast cells (r = -0.463, *P* < 0.001), Th17 cells (r = -0.369, *P* < 0.001), Tfh (r = -0.336, *P* < 0.001), CD8^+^ T cells (r = -0.275, *P* < 0.001), and NK cells (r = -0.241, *P* < 0.001) were negatively correlated with the MTFR2 expression (**Figure S3**). Then we verified the association between MTFR2 and Th2 cells. Th2 cells were featured with GATA3 expression. We examined the protein levels in 40 paired NSCLC tissue samples. Among 40 randomly selected pairs of tissues, 29 pairs showed upregulated MTFR2 and GATA3 expression. Five cases presented opposite MTFR2 and GATA3 expression in NSCLC tissues (**Figure 6C**). In summary, these findings indicated a positive connection between MTFR2 and Th2 cell infiltration levels in NSCLC tissue, indicating that MTFR2 may also promote LUAD progression *via* regulating Th2-cell function.

## Discussion

As far as we know, cancer metabolism depends on ATP productions by mitochondria through oxidative phosphorylation (OXPHOS) (22–24). Cellular functions include cancer growth, migration, and energy metabolism, relying on mitochondrial homeostasis (22, 25). In addition, mitochondrial fusion and mitochondrial fission are crucial processes that shape the mitochondrial network. Studies have shown that many mitochondrial fission proteins participate in the cell cycle, apoptosis, cell proliferation, and cell migration (26–28). Abnormal mitochondrial fission can lead to tumor carcinogenesis and development (11).

Mitochondrial fission regulator 2 (MTFR2), also known as family with sequence similarity 54 member A, promotes mitochondrial fission (29). Published studies have also proven that MTFR2 plays an essential role in switching OXPHOS to glycolysis, activating the signaling pathway that promotes cell proliferation, invasion, and migration in oral squamous carcinoma and breast carcinoma (15, 16). Another study showed that MTFR2 could regulate tumor genesis, drug resistance, and tumor recurrence in glioma by activating TTK signaling (12). In breast cancer, MTFR2 expression was related to HER2 status and indicated a poor breast cancer prognosis (14). Based on the results of these studies, MTFR2 is closely associated with cancer. Moreover, in LUAD, only limited bioinformatics showed that MTFR2 expression was increased and correlated with sex, age, smoking history, neoplasm staging, histological subtype, and TP53 mutation status in patients. However, it lacks tissue sample validation. In addition, the role of MTFR2 and the underlying potential mechanism in LUAD have not been thoroughly studied.

In this study, we found that MTFR2 expression was significantly overexpressed in LUAD and a high expression of MTFR2 was associated with advanced TNM stages and poor survival in LUAD patients, which suggested that MTFR2 may be a tumor promoter in LUAD. To further investigate the function of MTFR2 in LUAD, GSEA analysis was performed and found that the mitotic phase and G2/S phase in the cell cycle were enriched in the MTFR2 high-expression group, implying that MTFR2 may be involved in LUAD cell proliferation. Next, we conducted cellular experiments to verify the above bioinformatical analysis. CCK-8 and transwell assay consistently showed that knockdown of MTFR2 expression could inhibit cell proliferation and metastasis in A549 and H1299 cell lines. Further flow cytometry assay showed that inhibition of MTFR2 can induce cell cycle G2/M phase arrest and G0/G1 phase reduction. In addition, SC-79 as an activator can stimulate the AKT pathway, which reverses the knockdown effect of MTFR2. Such results further proved that MTFR2 could promote cell proliferation *via* AKT signaling pathways.

The relationship between MTFR2 and immune cells has not been explored in cancers. Our study showed that MTFR2 expression was positively correlated with Th2 cell, γδ T cell, NK CD56^dim^ cell, T helper cell, aDC, and Treg infiltration. We found that mast cells, NK cells, and cytotoxic cells decreased in the MTFR2-overexpressed group. More importantly, we found that Th2 cells showed the strongest positive correlation with MTFR2 expression. The Th2/Th1 ratio was higher in early-stage lung adenocarcinoma and is considered to be involved in the progression of lung adenocarcinoma (30). Th2 cell infiltration is also related to drug resistance (31). These findings suggested that Th2 cells were negatively regulated immune cells in lung adenocarcinoma. Moreover, our results showed a positive correlation between MTFR2 expression and Th2-cell infiltration levels, implying that MTFR2 overexpression in LUAD patients may trigger pro-tumor immune responses. However, further studies are needed to confirm this correlation between MTFR2 and immune cells.

Although our study is the first to identify the role of MTFR2 in lung adenocarcinoma, there are still some limitations. First, how MTFR2 functions in mediating LUAD proliferation and metastasis and the related signaling pathways should be explored in future studies; furthermore, we only proved that MTFR2 could take part in LUAD proliferation and metastasis. Whether it can contribute to the modification of the tumor microenvironment, drug resistance to EGFR-TKI, or immunotherapy remained to be discovered.

In conclusion, for the first time, we proved that MTFR2 is overexpressed in LUAD tissues and associated with TNM stages and poor survival for patients. We also clarified that MTFR2 could promote LUAD cell proliferation and metastasis *via* the AKT pathway. In addition, it is significantly associated with Th2 immune cell infiltration in LUAD, suggesting that MTFR2 may be regarded as a potential prognostic indicator and therapeutic target for LUAD patients.

## Data Availability Statement

The original contributions presented in the study are included in the article/**Supplementary Material**. Further inquiries can be directed to the corresponding authors.

## Ethics Statement

Written informed consent was obtained from the individual(s) for the publication of any potentially identifiable images or data included in this article.

## Author Contributions

Idea and design: ZL, WD, PP. Development of methodology: YZ. Acquisition of data: ZL, WD, PP. Analysis and interpretation of data: ZL, WD, PP, JZ. Writing, review, and/or revision of the manuscript: ZL, WD. Administrative, technical, or material support: ZL, WD, PP. All authors contributed to the article and approved the submitted version.

## Funding

This work was supported by grants from the Projects of Medical Science and Technology Development Plan of Taicang City (Tc2018JCYL12) and the Natural Science Foundation of Jiangsu Province (Grant No. BK20210090).

## Conflict of Interest

The authors declare that the research was conducted in the absence of any commercial or financial relationships that could be construed as a potential conflict of interest.

## Publisher’s Note

All claims expressed in this article are solely those of the authors and do not necessarily represent those of their affiliated organizations, or those of the publisher, the editors and the reviewers. Any product that may be evaluated in this article, or claim that may be made by its manufacturer, is not guaranteed or endorsed by the publisher.

## Supplementary Material

Supplementary Figure 1 The forest plot indicated that MTFR2 was an indepent worse prognosis factor *via* unltivariate **(A)**, multivariate **(B)** and nomogram analyses **(C)**.

Supplementary Figure 2 GSEA analysis showed cellular responses to external stimuli, signaling by RHO GTPASE, infectious disease, M phase, DNA replication, transcription regulation by P53, and translation, cellular response to stress **(A–H)**.

Supplementary Figure 3 MTFR2 over-expression was positively associated with the Tgd cells **(A)**, NK CD56 dim cells **(B)**, T help cells **(C)** and aDCs **(D)**, while was significantly negatively associated with the mast cells **(E)**, Th17 cells **(F)**, Tfh cells **(G)**, NK cells **(H)** and CD8 T cells **(I)**.
