# Supplementary figures and images for: Prognostic Value and Potential Mechanism of MTFR2 in Lung Adenocarcinoma
Source: Front Oncol. 2022 May 5;12:832517. doi: 10.3389/fonc.2022.832517 (PMC9117628; doi:10.3389/fonc.2022.832517)

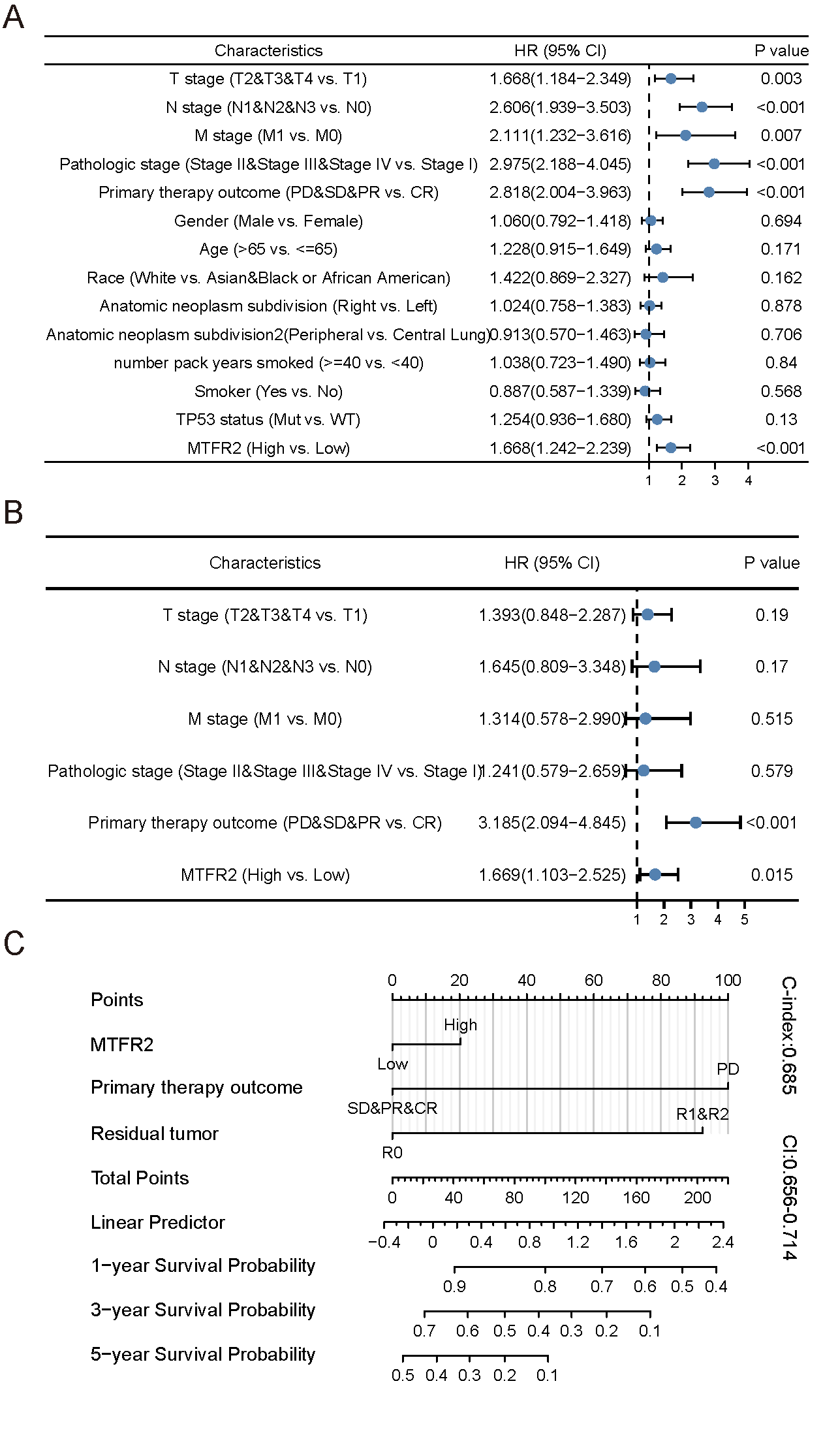

Supplement: Supplementary Figure 1 — The forest plot indicated that MTFR2 was an indepent worse prognosis factor via unltivariate (A), multivariate (B) and nomogram analyses (C). [file Image_1.tif]

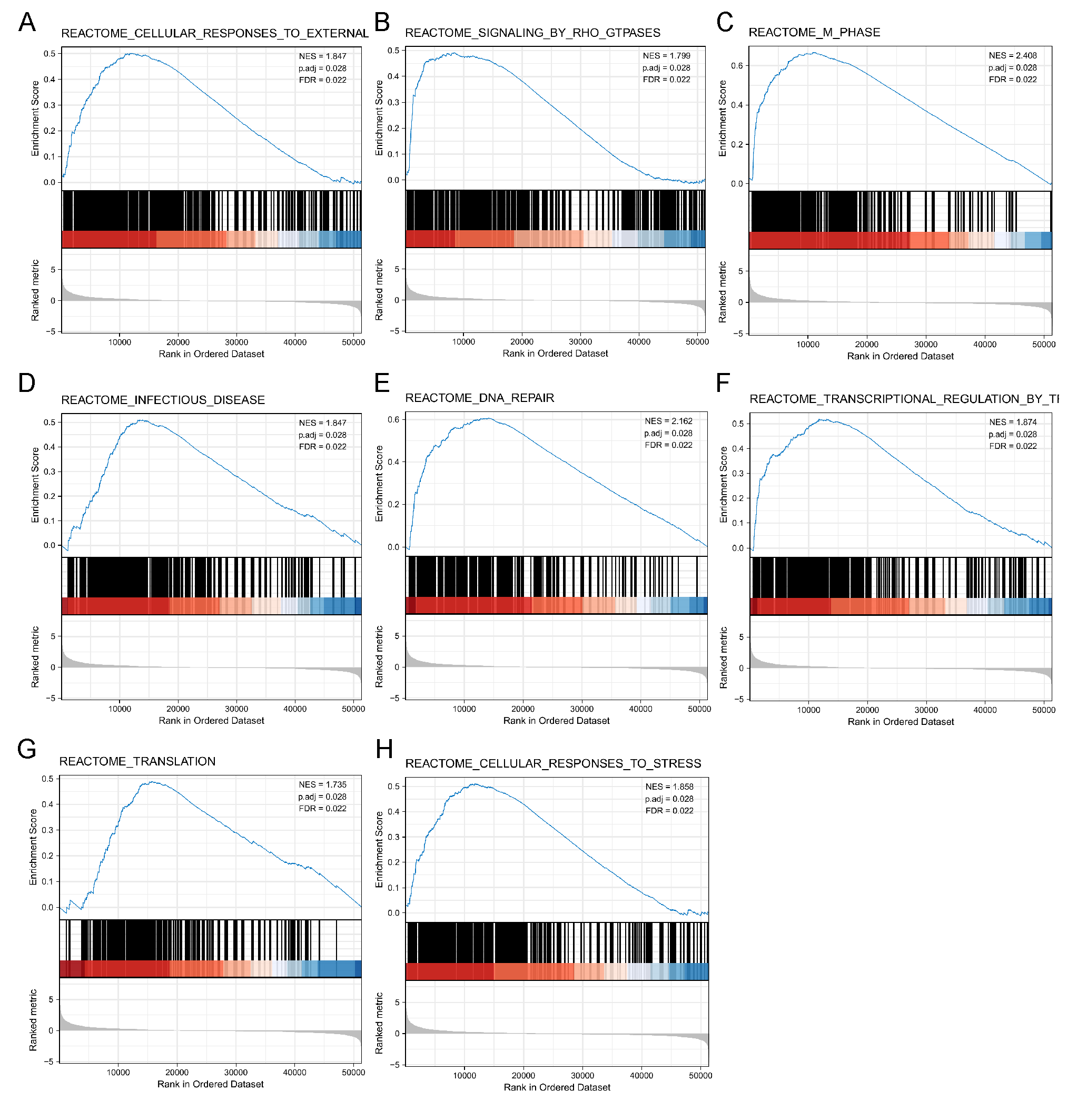

Supplement: Supplementary Figure 2 — GSEA analysis showed cellular responses to external stimuli, signaling by RHO GTPASE, infectious disease, M phase, DNA replication, transcription regulation by P53, and translation, cellular response to stress (A–H). [file Image_2.tif]

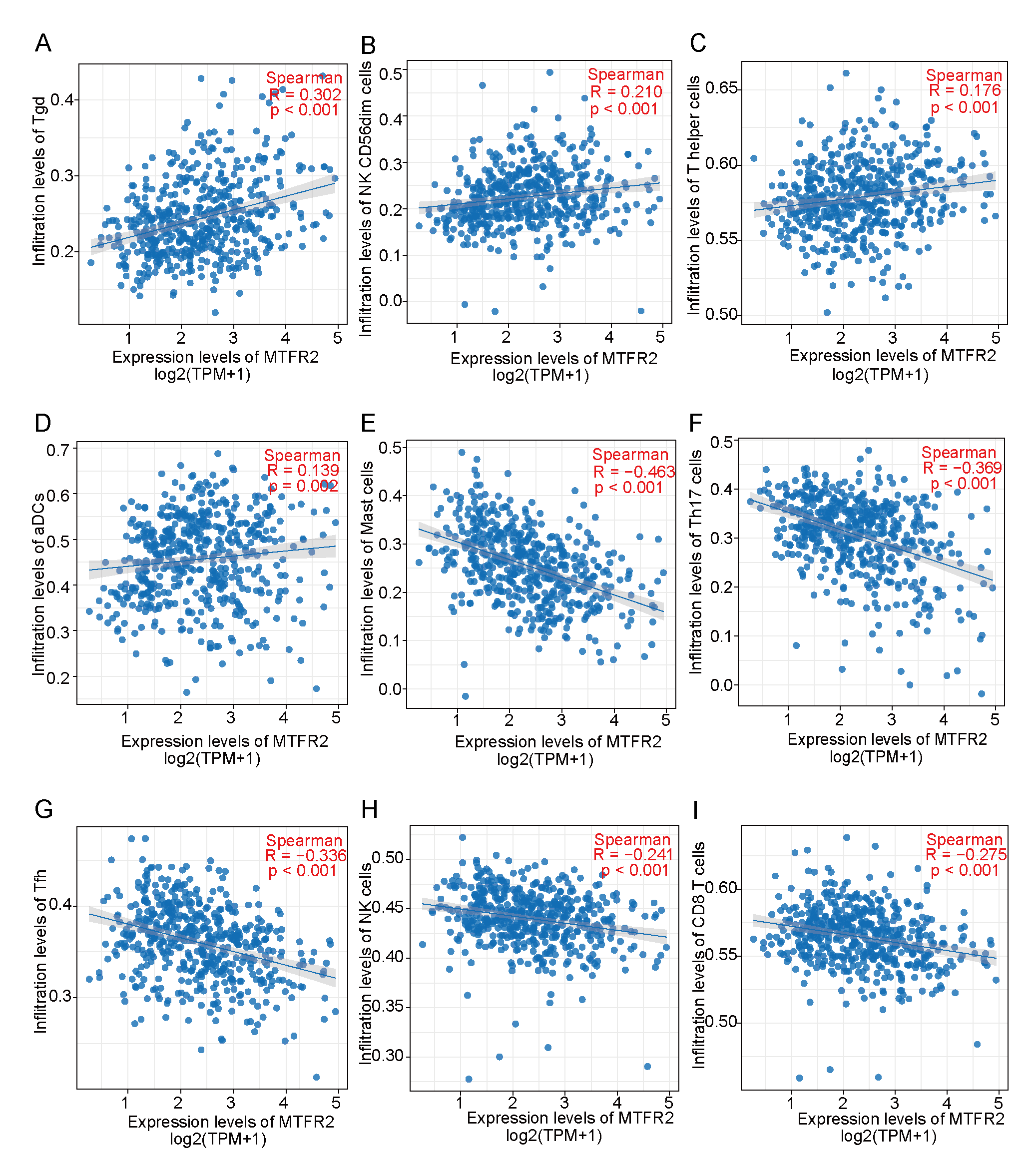

Supplement: Supplementary Figure 3 — MTFR2 over-expression was positively associated with the Tgd cells (A), NK CD56 dim cells (B), T help cells (C) and aDCs (D), while was significantly negatively associated with the mast cells (E), Th17 cells (F), Tfh cells (G), NK cells (H) and CD8 T cells (I). [file Image_3.tif]
