# Supplementary material for: Prognostic Value and Potential Mechanism of MTFR2 in Lung Adenocarcinoma
Source: Front Oncol. 2022 May 5;12:832517. doi: 10.3389/fonc.2022.832517 (PMC9117628; doi:10.3389/fonc.2022.832517)
Supplement: Supplementary file 4 [file Table_1.docx]

| **Table S1 Clinical characteristics of lung adenocarcinoma in TCGA** | | | |
| --- | --- | --- | --- |
| **Characteristics** |  | **Number of cases** | **Ratio(%)** |
| Topography(T) | T1 | 168 | 32.9 |
|  | T2 | 276 | 54.1 |
|  | T3 | 47 | 9.2 |
|  | T4 | 19 | 3.7 |
| Lymph node(N) | N0 | 330 | 65.9 |
|  | N1 | 95 | 19 |
|  | N2 | 74 | 14.8 |
|  | N3 | 2 | 0.4 |
| Metastasis(M) | M0 | 344 | 93.2 |
|  | M1 | 25 | 6.8 |
| Pathologic stage | Stage I | 274 | 54.3 |
|  | Stage II | 121 | 24 |
|  | Stage III | 84 | 16.6 |
|  | Stage IV | 26 | 5.1 |
| Primary therapy outcome | CR | 315 | 73.9 |
|  | PD | 68 | 16 |
|  | PR | 6 | 6 |
|  | SD | 37 | 8.7 |
| Gender | Female | 276 | 53.8 |
|  | Male | 237 | 46.2 |
| Race | Asian | 7 | 1.6 |
|  | Black | 52 | 11.6 |
|  | White | 387 | 86.8 |
| Anatomic neoplasm subdivision | Left | ­­­­­ 199 40 | |
|  | Right | 299 | 60 |
|  | Central Lung | 62 | 32.8 |
|  | Peripheral Lung | 127 67.2 | |
| Smoker | No | 74 | 14.8 |
|  | Yes | 425 | 85.2 |
| TP53 status | Mut | 241 | 47.4 |
|  | WT | 267 | 52.6 |
| KRAS status | Mut | 139 | 27.4 |
|  | WT | 369 | 72.6 |
