# Supplementary material for: Prognostic Value and Potential Mechanism of MTFR2 in Lung Adenocarcinoma
Source: Front Oncol. 2022 May 5;12:832517. doi: 10.3389/fonc.2022.832517 (PMC9117628; doi:10.3389/fonc.2022.832517)
Supplement: Supplementary file 5 [file Table_2.docx]

| **Table S2 Correlation between MTFR2 expression and clinicopathologic characteristic** | | | | |
| --- | --- | --- | --- | --- |
| Clinical characteristic | level | MTFR2 expression | | P value |
|  |  | Low | High |  |
| T stage (%) | T1 | 105(41.0%) | 63(24.8%) | 0.001 |
|  | T2 | 119(46.5%) | 157(61.8%) | |
|  | T3 | 22(8.6%) | 25(9.8%) |  |
|  | T4 | 10(3.9%) | 9(3.5%) |  |
| N stage (%) | N0 | 179(71.9%) | 151(59.9%) | 0.01 |
|  | N1 | 43(17.3%) | 52(20.6%) |  |
|  | N2 | 27(10.8%) | 47(18.7%) |  |
|  | N3 | 0(0.0%) | 2(0.8%) |  |
| M stage (%) | M0 | 173(96.6%) | 171(90.0%) | 0.012 |
|  | M1 | 6(3.4%) | 19(10.0%) |  |
| Pathologic stage (%) | Stage I | 154(61.1%) | 120(47.4%) | 0.005 |
|  | Stage II | 56(22.2%) | 65(25.7%) |  |
|  | Stage III | 35(13.9%) | 49(19.4%) |  |
|  | Stage IV | 7(2.8%) | 19(7.5%) |  |
| Primary therapy outcome (%) | CR | 167(76.6%) | 148(71.2%) | 0.108 |
|  | PD | 26(11.9%) | 42(20.2%) |  |
|  | PR | 3(1.4%) | 3(1.4%) |  |
|  | SD | 22(10.1%) | 15(7.2%) |  |
| Gender (%) | Female | 158(61.5%) | 118(46.1%) | 0.001 |
|  | Male | 99(38.5%) | 138(53.9%) | |
| Race (%) | Asian | 2(0.9%) | 5(2.3%) | 0.249 |
|  | Black or African American | 23(10.0%) | 29(13.5%) |  |
|  | White | 206(89.2%) | 181(84.2%) | |
| Anatomic neoplasm subdivision (%) | Left | 100(40.0%) | 99(39.9%) | 1 |
|  | Right | 150(60.0%) | 149(60.1%) | |
| Anatomic neoplasm subdivision2 (%) | Central Lung | 33(37.5%) | 29(28.7%) | 0.217 |
|  | Peripheral Lung | 55(62.5%) | 72(71.3%) |  |
| Smoker (%) | No | 43(17.3%) | 31(12.4%) | 0.132 |
|  | Yes | 206(82.7%) | 219(87.6%) | |
| TP53 status (%) | Mut | 67(26.3%) | 174(68.8%) | <0.001 |
|  | WT | 188(73.7%) | 79(31.2%) |  |
| KRAS status (%) | Mut | 79(31.0%) | 60(23.7%) | 0.082 |
|  | WT | 176(69.0%) | 193(76.3%) | |
| Age (%) | <=65 | 106(42.6%) | 132(53.9%) | 0.015 |
|  | >65 | 143(57.4%) | 113(46.1%) | |
| number pack years smoked (%) | <40 | 95(57.6%) | 79(42.5%) | 0.005 |
|  | >=40 | 70(42.4%) | 107(57.5%) | |
