# Supplementary material for: Prognostic Value and Potential Mechanism of MTFR2 in Lung Adenocarcinoma
Source: Front Oncol. 2022 May 5;12:832517. doi: 10.3389/fonc.2022.832517 (PMC9117628; doi:10.3389/fonc.2022.832517)
Supplement: Supplementary file 6 [file Table_3.docx]

| **Table S3 Association between MTFR2 expression and clinical pathological characteristics (logistic regression)** | | | |
| --- | --- | --- | --- |
| Clinical characteristics | Total(N) | Odds Ratio in MTFR2 expression | P-value |
| T stage (T2&T3&T4 vs. T1) | 510 | 2.11(1.45-3.09) | <0.001 |
| N stage (N1&N2&N3 vs. N0) | 501 | 1.71(1.18-2.49) | 0.005 |
| M stage (M1 vs. M0) | 369 | 3.20(1.32-8.97) | 0.015 |
| Pathologic stage (Stage II &Stage III &Stage IV vs. Stage I) | 505 | 1.74(1.22-2.48) | 0.002 |
| Primary therapy outcome (PD&SD&PR vs. CR) | 426 | 1.33(0.86-2.05) | 0.201 |
| TP53 status (Mut vs. WT) | 508 | 6.18(4.23-9.14) | <0.001 |
